# Kawasaki Disease in Infants in the First 3 Months of Age in a Mexican Population: A Cautionary Tale

**DOI:** 10.3389/fped.2020.00397

**Published:** 2020-07-21

**Authors:** Luis Martín Garrido-García, Juan Humberto Gutiérrez-Alanis, Ana Isabel Ramírez-Perea, Adriana Tremoulet, Marco Antonio Yamazaki-Nakashimada

**Affiliations:** ^1^Department of Cardiology, National Institute of Pediatrics, Mexico City, Mexico; ^2^Department of Pediatrics, National Institute of Pediatrics, Mexico City, Mexico; ^3^Department of Pediatrics, University of California San Diego/Rady Children's Hospital, San Diego, CA, United States; ^4^Department of Immunology, National Institute of Pediatrics, Mexico City, Mexico

**Keywords:** Kawasaki disease, infants, cardiac complications, giant coronary artery aneurysms, Latinamerica

## Abstract

**Background:** Kawasaki disease (KD) is an acute febrile illness that largely affects young children before 5 years of age. Younger children with KD are reported to have a higher prevalence of coronary artery abnormalities. Little is known about infants in the first 3 months of age diagnosed with KD.

**Methods:** A retrospective study was conducted at the National Institute of Pediatrics in Mexico City from 1995 to 2019. Clinical features, laboratory results and cardiac outcomes were recorded. Infants in the first 3 months of age were compared with older patients. Wilcoxon-Mann-Whitney analysis was performed for continuous variables and Fisher's exact test for categorical variables.

**Results:** Six hundred and eighty-eight cases of KD were included in this study. Fourteen cases were diagnosed in the first three months of age. Heart failure and KD shock-syndrome was found in five cases (35.7%). Giant coronary artery aneurysms were found in six cases in the younger group (42.9%).

**Conclusions:** Diagnosis of KD in children younger than 3 months of age is rare. In most cases, an incomplete presentation contributed to a delay diagnosis, treatment, and complications. Younger patients with KD have an increased risk of presenting cardiac complications, including giant coronary artery aneurysms, shock, and death.

## Introduction

Kawasaki disease (KD) is an acute febrile illness that largely affects young children with most cases diagnosed before 5 years of age. It is characterized by systemic vasculitis and it is complicated by coronary artery abnormalities (CAA).

Actually KD is considered the leading cause of acquired heart disease in children in developed countries (1). The highest incidence rate of KD in Japan is reported in children between 9 and 11 months of age (2). Younger children with KD are reported to have a higher prevalence of delayed diagnosis and therefore cardiac complications including CAA (3). In Mexico, as well as in other Latin-American countries, the incidence of the disease has not been reported.

The purpose of this study was to describe the clinical characteristics, laboratory results, treatments used and cardiac complications in patients in the first 3 months of age with KD and compared them with older children with KD in a Mexican population.

## Materials and Methods

A retrospective study was conducted at the National Institute of Pediatrics, which is an academic tertiary reference center in Mexico City, Mexico. The clinical records of patients under 18 years-old who were diagnosed in the acute phase of KD between August 1995 and August 2019 were retrospectively reviewed. The diagnosis of KD was made according the 2017 criteria of the American Heart Association (AHA). Those who had fever for 5 days and met more than four of the major criteria were classified as complete KD. As per the AHA guidelines, incomplete KD was defined as unexplained fever for 5 days and more, associated with two or three classical features of KD with coronary involvement (1).

Clinical and laboratory findings, in addition to echocardiographic measurements were recorded. Age, sex, weight, height, days of illness at admission, fever, presence of changes in lips, and oral cavity, changes in palms or soles, polymorphous exanthema, bulbar conjunctival injection, cervical lymphadenopathy, BCG reactivation, as well as gastrointestinal or neurological symptoms were recorded. We defined neurological manifestations if the patients presented with extreme irritability, somnolence, or encephalopathy, and seizures. Kawasaki disease shock syndrome was established if patients presented with cardiovascular collapse and hypotension during the acute phase of KD.

*Z*-score adjusted hemoglobin levels, white blood cells and platelets counts, neutrophil proportion, concentrations of C-reactive protein, erythrocyte sedimentation rate, albumin, sodium, and levels of bilirubin, aspartate aminotransferase and alanine aminotransferase were documented.

In all patients, an echocardiogram was performed at the time of diagnosis following the AHA recommendations. A pediatric cardiologist experienced in KD reviewed all echocardiograms corrected and adjusted the coronary dimensions by *z*-score body-surface area using the Haycock formula (4). Coronary artery dimensions were measured from the maximal diameter of the left main coronary artery, proximal left anterior descending artery, circumflex artery, and proximal right coronary artery and *z*-score was adjusted using the Dallaire's formula (5).

Treatment with intravenous immunoglobulin was used in patients with KD diagnosis. IVIG dose from 1995 to 1999 was 400 mg/kg/day for 5 days and after the year 2000 we used a single IVIG dose of 2 gr/kg.

Patients with incomplete records, without laboratory results or without echocardiography at diagnosis were excluded from the study.

Patients were divided in two groups by age at diagnosis. Group 1 children diagnosed with KD in the first 3 months of age and group 2 children with KD older than 3 months of age. Clinical manifestations, laboratory results, echocardiographic findings, treatment, and outcome were compared between these two groups.

Statistical analysis was performed using the SPSS program, version 21 (SPSS, Chicago IL, USA). Continuous variables were expressed as mean ± standard deviation and median; a Fisher's exact test was performed to compare categorical variables. Wilcoxon-Mann-Whitney test was used for normally distributed continuous variables; *p* < 0.05 were considered to be significant and the confidence interval was 95%.

The Institutional Research and Ethics Committee approved the study.

## Results

During the study period, 754 cases of KD were diagnosed at our institution; 66 cases were eliminated from the analysis because of incomplete data. We evaluated 688 cases of KD for this study. Fourteen of these cases were in the first 3 months of age (2.03%). Ten of these cases were male (66.6%). Median age at diagnosis in patients younger than 3 months of age was 2 months; the youngest patient with KD diagnosed at our institution started with fever at 15 days of age and was diagnosed 18 days later.

Time between the onset of fever and diagnosis of KD in children in the first 3 months of age was 16.86 ± 9.38 days compared to 8.66 ± 5.43 days in older patients (*p* < 0.001).

Regarding the classical signs of KD in patients younger than 3 months, in order of frequency were oral changes in 12 cases (85.7%) compared to 629 cases of older children (93.3%) (*p* < 0.196); exanthema in 11 cases (78.6%) compared to 577 (85.6%) (*p* < 0.768); changes in extremities in 10 cases (71.4%) compared to 492 (72.9%) (*p* < 0.768); conjunctival hyperemia was found in nine cases (64.2%) compared to 607 cases of older children (90.0%) (*p* < 0.007). The least frequent of the classical signs was cervical lymphadenopathy and was only found in five cases (35.7%) compared to 378 cases of older children (56.0%) (*p* < 0.172). Incomplete KD was diagnosed in six cases (42.9%) compared to 133 of older children (19.7%) (*p* < 0.046).

Other clinical signs found in our patients were BCG scar reactivation in six of our younger patients (42.9%) compared to 196 cases in older children (29.0%) (*p* < 0.380). Heart failure and Kawasaki disease shock-syndrome (KDSS) was diagnosed in five of the younger patients. Neurological manifestations were found in five cases (35.7%) compared to 83 cases of older children (12.3%) (*p* < 0.001). One patient developed peripheral vasculitis with distal gangrene of the right foot. The complete analysis of the clinical manifestations in patients in the first 3 months of age and the comparison to older children is shown in Table 1.

**Table 1:** Comparison of complete clinical manifestations in patients younger and older than 3 months of age in the acute phase of Kawasaki disease in a population of Mexican children.

**KD**					
***n*** **= 688**					
**Clinical manifestation**	**First 3 months**		**>3 months**		***p-value***
	***n*** **= 14**		***n*** **= 674**		
	**No**	**%**	**No**	**%**	
Male	10	71.4	441	65.4	0.781
**Days to diagnosis**	**Median 16**		**Median 7**		**<0.001**
	**Range (4–32)**		**Range (3–41)**		
Oral changes	12	85.7	629	93.3	0.196
Exanthema	11	78.6	577	85.6	0.419
Changes in extremities	10	71.4	492	73.0	0.768
**Conjunctival hyperemia**	**9**	**64.2**	**607**	**90.0**	**<0.007**
Cervical lymphadenopathy	5	35.7	378	56.0	0.172
**Incomplete KD**	**6**	**42.9**	**133**	**19.7**	**<0.046**
BCG scar reactivation	6	42.9	196	29.0	0.380
**KDSS**	**5**	**35.7**	**37**	**5.5**	**<0.001**
Hospitalization days	Median 9		Median 5		0.116
	Range (1–23)		Range (0–60)		

*KD, Kawasaki disease; BCG, Bacille Calmette-Guérin; KDSS, Kawasaki disease shock syndrome. The bold values were meant to highlight the variables with statistically significance*.

At diagnosis, patients in the first 3 months of age had statistically significant lower levels of albumin and higher leukocyte and platelet counts. The complete laboratory results are shown in Table 2.

**Table 2:** Complete laboratory results in patients with Kawasaki disease younger and older than 3 months of age in a Mexican population.

**KD**			
***n*** **= 688**			
	**First 3 months**	**> 3 months**	
	***n*** **= 14**	***n*** **= 674**	
**Laboratory result**	**Median**	**Median**	***p-value***
	**(Range)**	**(Range)**	
**Hemoglobin gr/dl**	**9.15**	**11.7**	**< 0.000**
	**(5.4–11.5)**	**(3.0–16.2)**	
Hemoglobin *z*-score	−1.22	−10.63	0.304
	(−15.38–2.88)	(−19.83–6.57)	
**Leukocyte count (mm**^**3**^**)**	**20,150**	**12,800**	**<0.004**
	**(7,500–43,200)**	**(5,300–48,400)**	
**Platelet count (mm**^**3**^**)**	**512,000**	**347,000**	**<0.003**
	**(135,000–1,200,000)**	**(160–1,350,000)**	
ESR (mm/hr)	48	49	0.623
	(12–63)	(2–95)	
CRP (mg/l)	9.97	6.70	0.370
	(2.71–16.0)	(0.05–32.0)	
Sodium (mEq/lt)	135.5	136	0.699
	(133–142)	(121–157)	
**Albumin (g/dl)**	**2.27**	**3.10**	**0.011**
	**(1.6–3.9)**	**(1.0–3.8)**	
Total bilirubin (mg/dl)	0.70	0.62	0.717
	(0.50–1.1)	(0.10–10.0)	
Direct bilirubin (mg/dl)	0.20	0.16	0.175
	(0.2–0.4)	(0.01–6.9)	
Indirect bilirubin (mg/dl)	0.30	0.43	0.173
	(0.3–0.8)	(0.01–5.3)	
Alkaline phosphatase (IU/l)	128	195	0.113
	(109–206)	(100–924)	
GGT (IU/l)	120	47	0.466
	(44–211)	(7–644)	
LDH (IU/L)	120	46	0.192
	(246–590)	(6–979)	
**AST (IU/l)**	**35**	**42**	**<0.050**
	**(16–226)**	**(11–696)**	
**ALT (IU/l)**	**26**	**39**	**<0.022**
	**(9–115)**	**(6–681)**	

*p-value < 0.05 indicates statistically significant*.

*ESR, Erythrocyte sedimentation rate; CRP, C-reactive protein; GGT, gamma glutamyl transferase; LDH, Lactate dehydrogenase; AST, aspartate aminotransferase; ALT, alanine aminotransferase. The bold values were meant to highlight the variables with statistically significance*.

**Figure 1:**
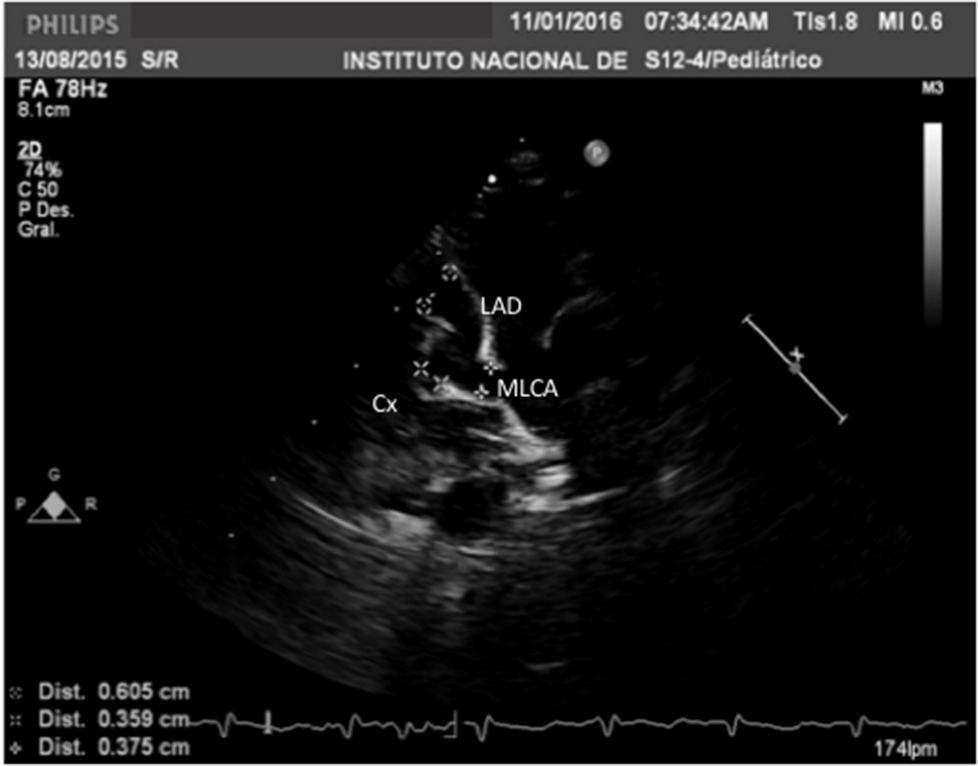
Echocardiogram in a modified long-axis parasternal view in a 2-months-old boy with Kawasaki disease diagnosed after 18 days of the onset of fever. Weight 4.7 kg, height 55 cm, BSA 0.27 m^2^. With a large coronary artery aneurysm in the main left coronary artery with z-score of +7.70; a giant coronary artery aneurysm in the left anterior descending artery with a *z*-score of +17.29 and a large aneurysm of the circumflex artery with a *z*-score of +8.99.

In the initial echocardiographic evaluation, myocarditis was found in four cases in the younger group (28.6%) compared to 81 cases (12.0%) of the older group (*p* < 0.061); pericardial effusion was diagnosed in six cases of the younger patients (42.9%) compared to 124 cases of the older group (18.4%) (*p* < 0.019). Pericarditis was diagnosed in six cases in patients in the first 3 months of age (42.9%) compared to 164 cases in the older group (24.3%) (*p* < 0.091). Coronary artery aneurysms were found in nine cases in the younger group (64.3%) compared to 195 cases in the older patients (28.9%) (*p* < 0.005). The comparison between the size of the coronary arteries and the adjusted *z*-scores in the 2 groups is shown in Table 3. In younger patients, giant coronary artery aneurysms (*z*-score > 10) were found in six cases (42.9%) compared to only 37 cases in the older group (5.5%) (*p* < 0.000) Figure 1.

**Table 3:** Comparison of coronary artery size and z-score adjustments in patients with Kawasaki disease in the first three months of age in a Mexican population.

**KD**					
***n*** **= 688**					
**Coronary size**	**First 3 months**		**> 3 months**		***p-value***
	***n*** **= 14**		***n*** **= 674**		
	***n***	**Median**	***n***	**Median**	
		**(Range)**		**(Range)**	
z-score left mean coronary artery	14	3.58	674	1.05	<0.002
		(0.11–21.91)		(−1.83–29.89)	
*z*-score left anterior descending artery	8	7.08	498	0.82	<0.010
		(−0.32–19.31)		(−1.66–26.46)	
*z*-score circumflex artery	8	5.04	473	0.65	<0.003
		(0.28–13.05)		(−2.15–17.59)	
*z*-score proximal right coronary artery	14	17.26	674	0.66	<0.002
		(0.76–21.09)		(−1.41–40.0)	

Intravenous immunoglobulin (IVIG) was used in 11 cases in the younger group (78.6%) compared to 624 cases of the older group (92.6%) (*p* < 0.062). Steroids were used in nine cases of the younger group (64.3%) compared to 313 cases (46.4%) (*p* < 0.167). A second dose of IVIG was given to two patients in the younger group (14.2%) compared to 43 cases of the older group (6.4%). No additional treatment were used in the younger patients. Two patients died in the acute stage of KD; one per group. An autopsy was performed in both patients which showed giant coronary artery aneurysms, thrombosis and acute myocardial infarction. The patient in the older group, thrombosis was also demonstrated in peripheral arteries (axillar, renal, and femoral arteries).

## Discussion

KD is the most common acquired heart disease in developed countries (1). In Mexico as well in other Latin American countries, KD is underdiagnosed and there are only isolated reports of the disease (6). However, the number of cases of KD in Mexico has been increasing over the years^1^ (7, 8).

KD is a disease of young children, with almost 80% of the cases diagnosed before 5 years of age (9). In the 23rd National Census of Kawasaki disease in Japan, Makino et al., found that the most frequent age of presentation was between 9 and 11 months of age (2). In the last Korean survey of KD, the most frequent age of presentation was 29 months (10). In both countries, which is where the highest incidence of KD exists, cases are rare in very young infants. In Mexico, according to national data from 2000 to 2018, the most frequent age of presentation of KD was 12 months, and these patients represented 27% of the diagnosed KD cases in Mexico^1^.

KD in very young children is a rare event. In a study in the San Diego area, between 2004 and 2013, Salgado et al. reported on 720 cases of the KD, in which 88 cases were in patients under the age of 6 months (3). Yoon et al. in Korea studied 239 cases of KD diagnosed between 2013 and 2015, and 26 cases were younger than 6 months of age (11). Singh et al. in a 20-years period study of KD in a single hospital in India, found 460 patients and only 17 cases were under 6 months of age (12). The largest study of KD in younger patients was performed in Korea by Lee et al., based on Korean National Surveys, 27,851 cases of KD were diagnosed and just 609 cases were children under the age of 3 months (2.18%) (13).

Our incidence of KD in infants in the first 3 months of age (2.03%) was similar to the large epidemiological studies in countries with a high incidence of KD.

As there is no accurate diagnostic marker for KD, diagnosis is made based on the combination of characteristic clinical findings. In very young children, a high incidence of incomplete and atypical KD has been reported (3, 11, 12). This clinical presentation often causes a delay in diagnosis and treatment and therefore infants with KD are at higher risk of developing severe cardiac complications as compared to older children (14). Maternal antibodies have been thought to be protective against KD supported by the rarity of KD before 3 months of age, contrasting with this theory are our findings and other multiples reports showing a severe presentation in this age group.

In our younger patients, classical clinical features of KD in order of frequency were oral changes (85.7%), exanthema (78.6%), changes in the extremities (71.4%), conjunctival hyperemia (64.2%), and cervical lymphadenopathy (36.7%). These data are similar with findings in younger patients with KD obtained in the San Diego area and in India (3, 12). Yoon et al. reported that conjunctival hyperemia was the most frequent clinical sign in patients under the age of 3 months which differed from our findings (11).

We found that six of our patients in the first 3 months of age had incomplete KD (42.9%), Salgado et al., found a 48.8% of incomplete presentation of the disease in patients under the age of 6 months (3). Hangai et al. based in the 22nd National Census of Kawasaki disease in Japan studied the presence of KD in newborns and found an incomplete presentation of the disease in 65% of the cases (15). Singh et al., in India studied 17 cases of KD in patients under 6 months of age and found that 88% of the cases had an incomplete presentation of the disease (12).

A BCG scar reactivation is an important clinical sign for diagnosis of KD in countries where the BCG vaccine is administered. Uehara et al., based in the 19th National Survey of Kawasaki disease in Japan, found that a BCG scar reactivation was present in 50% of patients with KD (16). This finding has also been reported by other authors like Singh, who found that a BCG scar reactivation was present in 11% of patients with KD younger than 6 months of age (12).

In a previous study performed in our hospital, Garrido-Garcia et al. found that a BCG scar reactivation occurred in 24.3% of 416 cases of KD, but this scar reactivation was increased to 32.3% in children younger than 12 months of age (17). In the present study, a BCG scar reactivation was found in six out of 14 cases in the first 3 months of age (42.9%). Araki et al. found that BCG erythema in patients with KD is most closely associated with the interval from the BCG vaccination to onset of KD (18). Intriguingly, some studies have found a correlation between BCG scar reactivation and severity of the disease (more inflammation with increased white blood cells, platelet counts, and transaminases) while others have not (18–20). As proposed by many authors, in countries where BCG vaccine is mandatory (including Mexico), a BCG scar reactivation could be a very important clue to diagnose KD in very young patients (18–25).

Another difference in the clinical picture of KD in very young patients is the increased frequency of atypical presentations including facial palsy, seizures, myocarditis, and cardiogenic shock (12, 23). In our study group, the atypical manifestations of KD were present in 35.7% of the cases compared to 10.7% of older patients (*p* < 0.019). The most commonly atypical manifestations found in our study were KDSS and severe central nervous system manifestations.

The incomplete and atypical presentations of KD in younger patients often causes a delay in diagnosis of the disease. In our study, we found an important delay in diagnosis, with 16.86 ± 9.38 days in younger patients, compared to 8.66 ± 5.43 days in older patients. This finding contrasts with other studies in countries with a greater incidence of KD, in which the diagnosis of KD is made in less than 10 days despite the age of the patient. Therefore, it is important to remember the AHA recommendations that any infant with unexplained fever for 7 or more days have an evaluation for KD, including an echocardiogram. Delay in the diagnosis with uncontrolled inflammation for more days probably contributed to the higher incidence of KDSS and CAA in our younger patients.

Yoon et al. reported that diagnosis of KD was made at 5.8 ± 2.0 days in younger patients, compared to 5.7 ± 1.5 days in older children (11). Also, Salgado et al. did not find differences in the time to diagnosis of KD with a median of 6 days both in children under 6 months and in older patients (3).

Until now, there is no specific diagnostic test of KD, but laboratory results reflect the degree of inflammation that is very common in young patients with KD. In our study, we found statistical significant increased leukocyte and platelet counts. These laboratory findings had also been confirmed by other groups (3, 9–12).

As almost all studies in younger patients, we found an increased frequency of coronary artery aneurysms in patients in the first 3 months of age, with 64.3% of our cases compared to 28.9% in older children. However, the most severe complication was the development of giant coronary artery aneurysms (42.9% compared to 5.5% in older patients). These findings were also confirmed by other groups who described patients with a late diagnosis of KD developing myocardial ischemia, circulatory collapse, and even death caused by coronary artery aneurysms and thrombosis (26, 27). Hangai et al. reported an incidence of coronary abnormalities in 17% of neonatal KD and in 16% in patients under the age of 6 months (15). Salgado et al. found that the presence of coronary lesions was 43.4% in younger patients compared to 19.5% in patients above the age of 6 months (3). Singh et al., reported up to 35% of coronary lesions in patients under the age of 6 months (12). In Taiwan, Chang found that coronary aneurysms developed in 65% in patients under the age of 6 months, compared to 19% in older patients (25).

**Figure 2:**
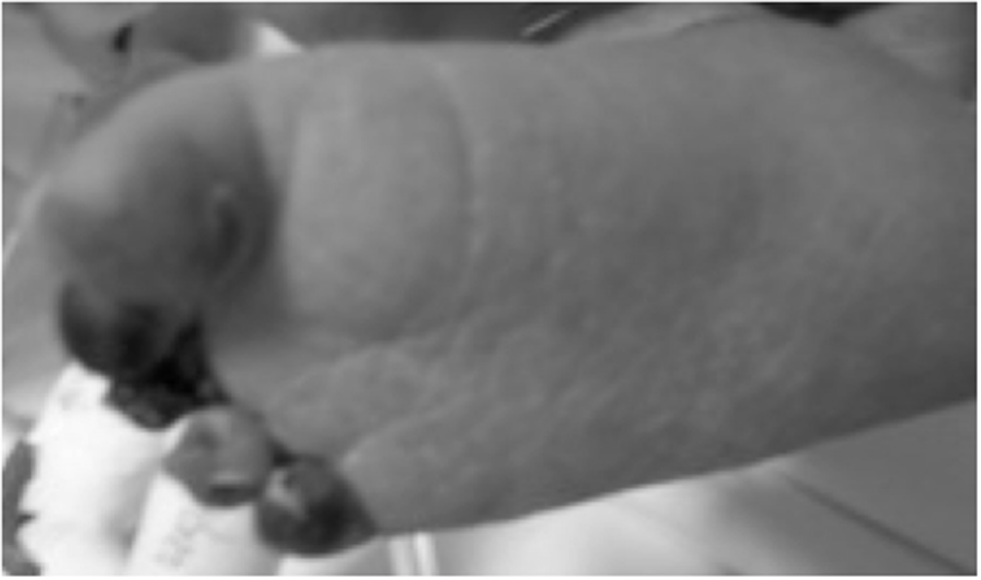
Peripheral vasculitis with distal gangrene in the right foot of a 45-days-old girl with Kawasaki disease diagnosed after 21 days of the onset of fever.

KDSS was particularly frequent in our younger patients. Peripheral gangrene is a complication of KD that occurs almost exclusively in infants younger than 7 months (28). One of our patients presented with peripheral vasculitis with distal gangrene in the toes of the right foot Figure 2. These observations reinforce the notion that KD is more severe in very young patients.

Finally, regarding appropriate treatment, in our study IVIG was administered in only 78.5% of the cases compared to 92.6% in older children. This finding was related to a delay in diagnosis, since patients in our younger group who did not receive IVIG, were patients who were not diagnosed in the acute phase of the disease and whose fever and systemic inflammation had resolved. In our younger group we also found a higher incidence of IVIG resistance (14.2%) compared to the older group (6.4%). This finding could be explained by an increased and persistent inflammation in younger patients.

## Limitations of the Study

Our study has strengths and limitations. To our knowledge, our series is the largest of KD in a single center in Latin America and is also the first to study KD in very young patients in the area. The most important limitation of the study is its retrospective design; therefore some date of our early patients are unknown. Data were obtained from a single institution, so it does not reflect the presentation of KD in the country. More studies have to be conducted in Mexico as well in other Latin American countries to establish the characteristics of infants with KD in Latin America.

## Conclusions

KD diagnosis in very young patients is difficult. KD should be suspected in any patient ≤3 months of age, with persistent high fever, without etiology, even if only some or none of the classic manifestations of KD are present. It is also important to note that a BCG scar reactivation may be a useful sign for early diagnosis and timely treatment in this group of patients in countries where the BCG vaccine is part of their immunization program. Finally, these patients have a higher rate of cardiovascular complications, atypical and incomplete presentations and KDSS as compared to older patients, and thus a high level of suspicion is critical.

## What's Known on This Subject

Kawasaki disease is the most common cause of acquired heart disease in developed countries; it is more common in children older than 1 year of age. An early diagnosis prevents the development of cardiac complications.

## What This Study Adds

Kawasaki disease in young infants, is an uncommon presentation with greater risk to develop cardiac complications. To our knowledge, this study represents the largest series of Kawasaki disease in very young infants in a Latin American country.

## Data Availability Statement

The datasets generated for this study are available on request to the corresponding author.

## Ethics Statement

The studies involving human participants were reviewed and approved by Ethics Committee National Institute of Pediatrics. Mexico. Written informed consent to participate in this study was provided by the participants' legal guardian/next of kin.

## Author Contributions

LG-G conceptualized and designed the study, performed the final data analysis, reviewed, and revised the final manuscript. JG-A designed the data collection instruments, collected data, carried out the initial analyses, and drafted the initial manuscript. AR-P designed the data collection instruments, collected data, carried out the initial analyses, and drafted the initial manuscript. AT reviewed the data analysis, reviewed, and revised the final manuscript. MY-N conceptualized and designed the study, reviewed, and revised the final manuscript. All authors approved the final manuscript as submitted and agree to be accountable for all aspects of the work.

## Conflict of Interest

The authors declare that the research was conducted in the absence of any commercial or financial relationships that could be construed as a potential conflict of interest.

## Footnotes

^1^Available online at: http://www.dgis.salud.gob.mx/contenidos/basededatos/BD_Cubos.html
